# Perceptions and experiences of community members on caring for preterm newborns in rural Mangochi, Malawi: a qualitative study

**DOI:** 10.1186/s12884-014-0399-6

**Published:** 2014-12-02

**Authors:** Austrida Gondwe, Alister C Munthali, Per Ashorn, Ulla Ashorn

**Affiliations:** Department of International Health, iLiNS Project, College of Medicine-Mangochi, University of Malawi and University of Tampere School of Medicine, Tampere, Finland; Centre for Social Research, Chancellor College, University of Malawi, Zomba, Malawi; Department of Paediatrics, Tampere University Hospital, University of Tampere School of Medicine, Tampere, Finland; Department of International Health, University of Tampere School of Medicine, Tampere, Finland

**Keywords:** Malawi, Preterm, Newborn care practices, Perceptions of preterm, Newborn community care

## Abstract

**Background:**

The number of preterm birth is increasing worldwide, especially in low income countries. Malawi has the highest incidence of preterm birth in the world, currently estimated at 18.1 percent. The aim of this study was to explore the perceived causes of preterm birth, care practices for preterm newborn babies and challenges associated with preterm birth among community members in Mangochi District, southern Malawi.

**Methods:**

We conducted 14 focus group discussions with the following groups of participants: mothers (n = 4), fathers (n = 6) and grandmothers (n = 4) for 110 participants. We conducted 20 IDIs with mothers to preterm newborns (n = 10), TBAs (n = 6) and traditional healers (n = 4). A discussion guide was used to facilitate the focus group and in-depth interview sessions. Data collection took place between October 2012 and January 2013. We used content analysis to analyze data.

**Results:**

Participants mentioned a number of perceptions of preterm birth and these included young and old maternal age, heredity, sexual impurity and maternal illness during pregnancy. Provision of warmth was the most commonly reported component of care for preterm newborns. Participants reported several challenges to caring for preterm newborns such as lack of knowledge on how to provide care, poverty, and the high time burden of care leading to neglect of household, farming and business duties. Women had the main responsibility for caring for preterm newborns.

**Conclusion:**

In this community, the reported poor care practices for preterm newborns were associated with poverty and lack of knowledge of how to properly care for these babies at home. Action is needed to address the current care practices for preterm babies among the community members.

## Background

Recent estimates indicate that there are 15 million preterm newborns annually and the number is increasing each year [1]. South Asia and sub-Saharan Africa accounts for almost two-thirds of the world’s preterm newborns and over three-quarters of the world’s newborn deaths due to preterm birth complications [1]. Worldwide almost half of preterm newborns are born at home and even for those born in facilities; essential newborn care (ENC) is often lacking [2]. Consequently, most of the preterm deaths occur at home in low-income and medium-income countries against a backdrop of poverty, sub-optimal care seeking and weak health systems [3]. It has been argued that most of these deaths would be averted by ensuring clean delivery, treating infections with antibiotics, promoting early and exclusive breastfeeding and keeping babies warm [4,5].

Malawi has the highest incidence of preterm birth in the world, estimated at 18.1 percent [1]. A previous study about preterm birth in rural communities in Malawi, described that neonatal and perinatal mortality was twice as high in preterm newborns as compared to term newborns [6]. Additionally, newborn survival interventions in Malawi such as care at birth and postnatal care have fewer improvements as compared to other interventions such as antenatal care and family planning [7]. Yet most newborn deaths happen in the first week and half of those in the first 24 hours of life [8] and among preterm newborns the lack of ENC may lead to death [9].

To our knowledge, there is a lack of evidence that people at community level in Malawi can easily care for preterm newborns. Information on local perceptions of preterm birth and care practices is important for programs aiming at improving preterm newborn health at the community level. Two studies in Malawi have previously reported on community concepts of preterm birth and knowledge of preterm birth respectively [10,11]. However, these studies did not investigate how preterm newborns were cared for. Thus, to improve newborn health outcomes in a resource poor setting currently challenged with a high incidence of preterm births [1], we undertook a qualitative study to explore the perceived causes of preterm birth among community members, recognition of preterm newborns, the reported care practices for preterm newborns and what people perceive as challenges in caring for preterm newborns at community level in Mangochi District in southern Malawi.

## Methods

We used a qualitative study design [12] to describe the community members’ perceptions about preterm birth and care practices for preterm babies. This was a sub-study of a randomized controlled trial testing the impact of lipid based nutrient supplements provided to women during pregnancy on birth outcomes. The randomized controlled trial was carried out in areas served by a government district hospital (Mangochi), two health centers (Lungwena and Namwera), and St Martin’s Hospital located in Malindi run by the Christian Health Association in Malawi (CHAM) [13]. The government district hospital is the major referral hospital in the district providing curative, preventive and maternal services.

### Sampling and recruitment procedures

Different sampling strategies were used for different groups of participants. Firstly, we used purposeful sampling for recruiting women, men, grandmothers, traditional birth attendants (TBAs) and traditional healers. We introduced the aim of the study to the local leaders in the community and engaged them to help in identifying potential participants to be included in the study. These local leaders were village heads in the catchment areas and because of their knowledge about the population in their communities; they were in a good position to locate the potential participants. For instance, we asked for men who were not yet grandfathers and had at least one infant at home born in the previous year and women who had given birth in the previous year. The criteria for selecting parents with an infant of less than one year was to explore their current care practices and experiences for newborn babies including their knowledge on the care for preterm babies. We included grandmothers who were staying with their grandchildren in the same household for focus group discussions (FGDs) because we assumed that they would have a greater role to support and advise the parents on how to care for a newborn baby including a preterm. We randomly selected the women who had given birth to a preterm newborn baby for in-depth interviews (IDIs) from the existing list of participants enrolled in the on-going randomized controlled trial to specifically explore the preterm care practices. Pregnancy outcomes for participants enrolled in the clinical trial were well documented and preterm newborn babies were defined as those born before 37 weeks gestational age.

### Data collection

Data collection took place between October 2012 and January 2013. Focus group discussions (FGDs) and in-depth interviews (IDIs) were used for data collection. A discussion guide was developed in English and translated into two local languages: *Chiyao*- the common local language in the catchment areas and *Chichewa* – the national language in Malawi. The same discussion guide was used for both FGDs and IDIs. Topics covered in the discussion guide were: perceived causes of preterm birth, definition of preterm birth, recognition of preterm newborns, care practices of preterm newborns and the associated challenges. We used IDIs mainly with TBAs, traditional healers and mothers of preterm newborns to get individual perspectives on the issues discussed. We conducted ten IDIs with mothers of preterm newborns, six TBAs and four traditional healers. We conducted a total of 14 FGDs, with a total of 110 participants from the following groups: women (four groups), grandmothers (four groups) and men (six groups). Each focus group consisted of 4 to 12 members. FGDs [14,15] were mainly conducted with grandmothers, fathers and mothers to explore their group views. We planned 5 focus groups with men, more than women focus groups based on the assumption that men are rarely involved in taking care of newborns. However, we conducted 6 FGDs with men instead of the planned 5, because in one site we received more participants than we could include in one group. None of the approached participants refused to take part in the interviews. A summary of the total number of interviews conducted is presented in Table 1.

**Table 1:** **Interview type**

	**n = 130**
FGD (women)	4 groups (49)
FGD (men)	6 groups (29)
FGD (Grandmothers)	4 groups (32)
In-depth interview with mothers to preterm babies	10
In-depth interview with TBAs	6
In-depth interview with traditional healers	4

The interviews were carried out in one of the local languages based on the interviewees’ preference. In FGDs, we encouraged all participants to contribute to the discussions and each session took approximately 40 to 50 minutes. The interviewers briefed the participants on the aim of the study. All the interviews were recorded digitally. One researcher with experience in conducting qualitative studies (AG) and two research assistants (moderator/note taker), fluent in both local languages conducted all the FGDs and IDIs. All the IDIs were held in participants’ homes and FGDs in either a school or a place selected by a group away from disturbances. All participants received two bars of soap to compensate for their time.

### Data management, analysis and ethical considerations

The recorded interviews were transcribed verbatim and then translated into English because some of the authors were not familiar with the local languages. The transcribed text was then compared with handwritten notes by the note-taker. We manually analyzed data using a content analysis approach [16]. The lead author read through the transcripts to get a sense of the whole text identifying themes, coding and assigning them to categories [17]. A summary of the findings was made, shared among the authors and agreed upon. We obtained oral and written consent from all the participants before the start of each interview. The College of Medicine Research Ethics Committee (COMREC) approved the study.

## Results

### Perceived causes of preterm birth

Participants reported a number of causes of preterm birth, which can be grouped into two aspects: maternal factors during pregnancy and general social factors as detailed in Table 2. The maternal factors included pregnant woman not eating good quality and enough food, pregnant woman doing excess household chores, husband beating his pregnant wife, frequent illnesses of the pregnant woman, having a previous abortion, history of preterm birth in the family, early and late childbearing. One male FGD participant noted that early childbearing was becoming more common in their communities, which many agreed to*.*

**Table 2:** **Summary on perceived causes of preterm birth in the community**

**Causes of preterm birth**	
Maternal factors	Frequent illnesses during pregnancy
	Pregnant woman overworking during pregnancy i.e. farming, fetching firewood
	Husband beating the pregnant wife
	Pregnant woman eating too little and poor quality food, i.e. always eating okra, sometimes sleeping without food
	Young and old maternal age
	The history of a preterm birth in the family
	Attempted abortion or previous abortion using traditional medicine
	Giving birth to many children
	Short intervals between births
	Pregnant woman delays in starting antenatal care
	Sexually transmitted infections (*matenda opatsirana pogonana*) i.e. syphilis (*chindoko*), gonorrhea (*chinzonono*) and HIV/AIDs
	Traditional diseases such as *Moto/tsempho* or *ndaka/nsanjiko*
	Malaria, epilepsy, high blood pressure, anemia
General social factors	Use of family planning methods, especially injections
	Will of God
	Witchcraft
	Use of local medicine during pregnancy

*“Nowadays girls as young as under 16 years have already started child bearing and they are too young and not physically developed. These girls usually give birth to preterm newborns because their body organs are not well developed”.***FGD, Men.**

Furthermore, during FGDs, several women reported that a woman having too many births, especially eight or more times was dangerous because the uterus was much utilized and could lead to preterm birth.

*“The uterus has weight but when you have given birth so many times, it loses the weight and becomes lighter (kupyapyala) failing to hold the pregnancy”.***FGD, Women.**

Participants further reported a number of illnesses during pregnancy that would lead to preterm birth, such as malaria, high blood pressure *(matenda a mtima),* anemia *(kuchepa magazi)*, asthma *(mphumu)* and epilepsy *(khunyu).* In addition, all participants emphasized that sexually transmitted infections (STIs) (*matenda opatsirana pogonana*) such as gonorrhea (*chinzonono*), syphilis (*chindoko*) and HIV/AIDS were the main cause, if left untreated as the quote below explains:

*“If a woman has a disease in the her womb and if it is transmitted to the baby inside the womb, then the baby doesn’t stay longer in the womb - move out of place, and eventually the baby comes out too soon before it is ripe. Most of these diseases are sexually transmitted diseases such as syphilis, gonorrhea and HIV/AIDs.***IDI, TBA.**

Participants also perceived traditional illnesses, i.e. *mwanamphepo* explained in Table 3 among women and *likango* for both women and men that would lead to a preterm birth or miscarriage. The participants also emphasized *moto*/*tsempho* in *Chichewa* language or *ndaka*/*nsanjiko* in *Yao* language that caused preterm birth because the pregnant woman carelessly shared utensils and ate food prepared by a woman who recently had a miscarriage, had a preterm birth and who has frequently engaged in sex with a man. Participants felt that a husband having a sexual relationship outside marriage causes miscarriage or preterm birth because there is contamination of blood through sexual intercourse of the husband with another woman outside marriage believed to be ‘hot’ which is then mixed with the blood of the pregnant wife through sex also believed to be ‘hot’. Participants further reported that a pregnant woman could also give birth to a preterm newborn if having sex outside marriage. The two quotes below help to explain more:

**Table 3:** **Definitions of vernacular terms related to perceived causes and care of preterm baby**

**Most common words**	**Meaning**
Mwanamphepo *(Chichewa/Chiyao*)	Traditional illness found in women believed to cause sores inside the womb and causes miscarriage or preterm birth
Likango (*Chichewa/Chiyao*)	Traditional illness found both in men and women. Causes preterm birth and also death to the newborn before the preterm newborn reaches one year. Can be cured by traditional medicine.
Ndaka/nsanjiko (*Chiyao*) or tsempho/moto (*Chichewa*)	Caused by sexual impurity if the husband of a pregnant woman is having sex outside marriage while the wife is pregnant.
	Sometimes caused by the pregnant woman herself if she is having sex with other men besides the husband
	Causes preterm birth if the pregnant woman carelessly eats or shares utensils with a woman who had miscarried before, had a preterm newborn or a woman who is engaging in sex
Kupyapyala (*Chichewa*)	A state of being weak and thin
Anamutembereza (*Chichewa*)	Expression of been cursed by someone

*“Some of the women could go and sleep with other men (nsanjiko) when their pregnancy is already tired and we happen to know such women because when delivering, they start by pouring out a lot of water and then delivers a preterm newborn …”***IDI, TBA.**

*“We know that the man had sex with another woman so that made the wife to lose the pregnancy because the blood from the other woman is different from the wife’s blood and sometimes the woman who slept with the husband had miscarried before”.***FGD, Men.**

The general social factors were heredity, the use of family planning methods, witchcraft and that the pregnant woman might not have spoken respectfully to elderly people who in turn cursed (*anamutemberedza*) her. Mothers with a previous preterm birth commonly reported that it was God’s will. However, one mother of a preterm newborn emphasized that use of the traditional medicine whilst pregnant would also cause a preterm birth because the system in the uterus ‘*is disturbed’*.

### Recognition of preterm newborns

All participants perceived preterm newborn as a baby who was born before the pregnancy had lasted for nine complete months. Participants reported that they counted the number of months from the last menstrual period as highlighted in the quote below:

“*If born at home and when we count months and find that the months are not adding up to 9 months, we know that the baby is born preterm and we take the baby to the hospital”.***FGD, Men.**

Furthermore, participants reported using physical features ‘see a summary of points on recognition of preterm newborns’ to recognize a preterm baby. Grandmothers and TBAs commonly reported that the skin of preterm newborn is too soft, has many wrinkles, is transparent and weak (*kupyapyala*) in the *Chichewa* language just like a piece of paper.

Summary on recognition of preterm babies

- Baby is too small, fails to breathe properly and have transparent lips which are soft
- Baby fails to breastfeed, sunken forehead and skin having many wrinkles
- Baby born with few hairs, have few eye lashes and nails not fully developed
- Body looks watery to show that it is not fully developed
- Looks malnourished and anemic
- Counting the number of the months if less than 9 months corrected gestational age
- Baby looks sick, has a pale body with pale teats and soft body scaring people to hold
- Fails to pass stool in the first day of life

### Care practices for preterm newborns

All participants emphasized that preterm newborns received extra care compared to term newborns because most of them could easily die if not well cared for. The majority of participants commonly reported providing warmth as the main method of care. It could be provided by wrapping the newborn with warm materials, making fire inside the house, closing windows, doors and keeping the baby inside the house all the time. Grandmothers and TBAs advised women to squeeze milk from their breasts and give it to the baby because preterm newborns could not suckle enough milk on their own. TBAs mainly reported encouraging mothers to preterm babies to use plastic bottles and bags with hot water inside as explained in the quote below:

*“Sometimes, we fill hot water into two plastic bottles, and a plastic bag. We lay the preterm newborn on top of the plastic bag and the two bottles are placed side by side of the newborn. In that way, the newborn is like still into her mother’s womb, feeling warm*”. **IDI, TBA.**

The majority of participants reported that the preterm newborn was not bathed until it reached 9 months corrected gestational age. Additionally, almost all women in the FGDs and IDIs reported that most families with preterm newborns did not allow other people to see the newborn until it had reached 9 months corrected gestational age as explained below.

*“We restrict the people who come to see the baby. We do not allow anyone to come and see the baby because we are afraid of those who could do something bad – such as bewitching the baby, the way they hold or carry the baby and some are coughing as well so we get scared that they would infect the baby. So we only allow those people within the family, but not anyone else”.***FGD, Women.**

A few women in the FGDS reported that care involved maintaining good hygiene i.e. keeping the house clean, washing the newborn clothes and sprinkling water on the house surroundings to control dust. The other reason for confining the preterm newborn indoors was that parents feared discouragements from other people as one of the mothers to preterm newborn babies explained in the quote below:

*“We just tell them that you are not going to see the baby because it is preterm. In a normal situation, it means it is not yet born. Because when you allow them, they start gossiping that the baby is too tiny and cannot survive”.***IDI, Mother of a preterm newborn.**

None of the participants reported using skin-to-skin kangaroo care for preterm newborns. When asked, some acknowledged to have heard of it from the radio but did not know how to do it. However, a mother of a preterm newborn said that she practiced it in the hospital, but at home, it was difficult because of several household chores. A summary of reported care practices is presented in Table 4.

**Table 4:** **Summary of care practices and challenges**

Care practice for preterm newborns	The preterm newborn is not bathed until it reaches 9 months corrected gestational age
	Keeping the baby inside the house until it reaches 9 months corrected gestational age
	Windows and doors of the house are kept closed all the time
	Maintaining a clean environment (washing newborn clothes and sprinkling water around the house to control dust)
	Use of plastic bottles and bags with hot water inside to provide warmth
	Make fire inside the house to keep the house warm
	Wrap the baby with blankets
	Expressing breast milk (mothers squeezing the breast milk into a cup and using a spoon to feed the newborn)
	Couples with a preterm newborn refrain from sex until required time when couples take traditional medicine
Challenges faced in caring for preterm newborns	The preterm newborns fall sick often times
	Poverty- no money to buy paraffin, to pay hospital bills and transport, to buy warm materials and to improve the condition of the house if leaking
	Mother to preterm baby fails to do business and household chores i.e. farming and fetching firewood
	Men start having other sexual affairs outside marriage
	Lack of knowledge on how to properly care for preterm newborns

### Challenges of caring for preterm newborns in the community.

Almost all participants reported that the care of a preterm newborn was demanding, requiring the mother to be available all the time, thereby affecting business, farming and household chores. Men reported that they did not take part in carrying the baby, but provided support by fetching firewood, paraffin (a liquid which is used for a lamp to give light at night), and relish (a dish that is served with the Malawian staple food - *nsima*). Paraffin helps the mother to constantly check on the newborn at night while fetching for the relish and firewood are duties that a woman normally does so that the family can have something to eat. The women agreed with this assessment stating that having a preterm newborn was a burden on women because men did not help much in caring for the newborn. The majority of women further complained that instead of helping, their husbands started seeing other women which disturbed their marriages. The quote below explains more about how a preterm newborn in the family influenced men having sexual partners outside marriage.

*“A preterm birth is considered like a miscarriage (chitayo); requiring the family to wait for the appropriate time to take local medicine for chitayo before the family resumes sex, failing to do so the husband can die and this waiting time forces some men to go for other women”.***FGD, Women.**

Participants reported challenges of poverty and lack of knowledge on caring for preterm newborns. Because of poverty, parents failed to buy warm materials, lived in cold houses with grass thatched leaking roofs and failed to rush the baby to the hospital because of lack of money for transport and to pay for the hospital bills. In one of the catchment areas, the nearest health facility was a pay for-service hospital and participants complained of lack of money to access the facility.

*“Because of lack of money when your child falls sick, you wait first so that you can do piece work to find money – poverty is a problem here and these kind of babies when they fall sick, it means you have to rush to the hospital but this doesn’t happen because the nearest hospital is a paying one”.***FGD, Men.**

All participants concurred that many preterm newborns had failed to survive because of lack of proper care in the homes. However, the majority of participants mentioned that they were able to tell that the preterm newborn was sick because the baby would develop a fever, not breath normally, change skin color to yellow, coughing and crying often times when the mother tries to hold the newborn. Grandmothers and TBAs said that they always provided traditional medicine to the preterm newborns before going to the hospital.

*“We find long queues at the hospital and a preterm baby can die whilst waiting to be attended so after giving it local medicine, we then decide to start off for the hospital because even if you go sometimes they tell you that there is no medicine”.***IDI, TBA.**

## Discussion

In our study, we used FGDs and IDIs to explore perceptions of preterm birth and care practices for preterm newborns among community members in the Mangochi district in southern Malawi. Some of the perceptions of preterm birth reported in our study, such as maternal factors are similarly reported as risk factors for preterm birth in previous qualitative and epidemiological studies in Malawi and elsewhere [10,18-21]. Tolhurst *et al.*, in a study on ‘perceptions of preterm birth in Malawi’ similarly reported witchcraft, sexual impurity, traditional illnesses such as *mwanamphepo* and *likango* as causes of preterm birth and miscarriages [10]. Although it is well known that early pregnancies have public health consequences for both the mother and the newborn, in our study areas participants reported that early childbearing was common and was associated with preterm birth. Similar findings were reported by 78% of the respondents in a study conducted in Dowa district of Malawi who were concerned that teenage pregnancies were becoming common in their area [22]. Furthermore, several studies have reported higher rates of preterm deliveries among adolescent mothers as compared to adults [23,24]. Additionally, we found that participants in our study perceived the use of family planning methods such as injections, use of traditional medicine whilst the woman is pregnant and previous abortion to cause preterm birth.

We found that the majority of participants reported the concept of providing warmth as the most common care practice for preterm newborns. Other studies also recommend that simple care practice such as provision of warmth to preterm newborns should be universal [9,25-27]. However, some reported practices of generating warmth in our study, especially use of plastic bottles with hot water inside could be dangerous to the newborn if not properly handled. In contrast, the use of skin to skin contact for providing warmth was not mentioned and the majority of participants when asked reported lack of knowledge about skin to skin kangaroo mother care. Another qualitative hospital based study in Uganda also reported that participants did not know why and how skin to skin care should be done [28]. Furthermore, we suggest that the period of delayed bathing (until the baby reaches 9 months of corrected gestational age) is over emphasized and there is a need for further research to investigate the exact period of delayed bathing. However, we did not find out if the delayed bathing was after the first bath or that the baby was not bathed at all. Another study has also reported lack of evidence as to how long to delay, especially if the bath can be warm and in a warm room [29]. In our study, keeping the preterm newborn baby indoors and not allowing other people outside family members to see the baby was the most reported care practice in order to protect the baby from infections and discouragement from other people. This was also reported in Tanzania, where families secluded both baby and mother for 40 days so as to protect the newborn from witchcraft [30]. Maintaining a clean environment of the preterm newborn, such as washing the newborn clothes, keeping the house and its surroundings clean was another care practice reported in this study.

Interestingly, we found that grandmothers encouraged mothers to squeeze milk from the breast and feed the newborn using a spoon and a cup in agreement with preterm newborn care recommendations [31]. However, none of the participants reported promoting early and exclusive breastfeeding for preterm newborns. The role of grandmothers in newborn feeding practices, including breastfeeding has also been reported in many studies [32-34]. Similarly, perceptions of sexual impurity around pregnancy and childbirth have been reported in some parts of Malawi and Bangladesh [10,34-37]. According to Bezner’s study in the northern part of Malawi, grandmothers and other elders of the village report that they would recognize if a child is suffering from *‘moto’* or has died of ‘*moto’* - a disease described traditionally to be associated with sexual impurity [34]. In Bangladesh, families similarly know that children are vulnerable and at increased risk for serious health problems during the first weeks of life and take action to protect them [38]. The similarities of these studies from Malawi and Bangladesh are that they both report on sexual impurity in relation to care practices for any newborn not specifically for preterm babies. However, the similarities of these findings across these two countries probably suggest that programs targeting newborn health should consider such deep rooted perceptions in order to be successful.

In the present study participants report diverse challenges, including economic and health system related issues such as the lack of medication in public facilities and long queues. Lack of money was cited as the major challenge as many families are reportedly poor and could not afford to properly care for the preterm newborns including taking them to the hospital in case of sickness. In one of the catchment areas, the nearest health facility is a pay for-service hospital and this delays decision making to access medical care for a sick preterm newborn. This finding is in line with what others have found [39,40]. Actually, it has been acknowledged that poverty undermines maternal, newborn and child health through numerous pathways, including reduced care-seeking and access to health care services [40,41]. In order to mitigate the impact of poverty on maternal and infant health, there is a policy in Malawi that allows free access to services at CHAM hospitals with a focus on maternal and neonatal interventions [42]. However, the CHAM hospital in the catchment areas was not yet a beneficiary at the time of data collection. Similar interventions are reported in India, where the government has introduced a program that entitles all pregnant women and neonates to free care at public facilities, including free drugs and free transport to and from home [36].

The strength of this study is that we triangulated the data by using both FGDs and IDIs to explore community perceptions and care practices of preterm newborns. In addition, we included male participants in our study in a society which specifies gender roles assuming that men are not expected to know much about caring for newborn babies. The limitation of our study is that we did not include grandfathers who may have different opinions on preterm birth and care practices. Furthermore, the findings of this study cannot be generalized to all communities in Malawi as the study was conducted among a few people within the catchment areas of an active clinical trial. Although the present study was conducted in catchment areas that have had multiple studies, we believe that the response bias was minimized because no similar study had been conducted before. In addition, some of the participants in our present study were not involved in the ongoing clinical trial. Another limitation is that we did not ask for a place of delivery, so those who gave birth at home might have different views from those who delivered in a health facility on care practices. Possibly those who delivered at the facility had different care practices. However, even when mothers deliver at the health facility, they are quickly discharged and most of the newborn care happens at home.

## Conclusions

In this community, the reported poor care practices to preterm babies were associated with many challenges such as poverty and lack of knowledge on how to properly care for these babies at home. There is a need for action to address the current care practices for preterm newborn babies among the community members in order to improve survival of the increasing numbers of preterm babies in Malawi. These findings add new knowledge to the existing literature on the preterm newborn care which can foster appropriate preterm newborn care practices in the rural communities.

## Abbreviations

TBA: Traditional birth attendant
CHAM: Christian Health Association in Malawi
IDIs: In-depth interviews
FGDs: Focus Group Discussions
MoH: Ministry of Health
ENC: Essential Newborn Care
STI: Sexually Transmitted Infections
WHO: World Health Organization
